# Bioefficacy of Zinc oxide nanoparticle synthesis and their Biological, Environmental applications from *Eranthemum roseum*

**DOI:** 10.1016/j.toxrep.2024.101758

**Published:** 2024-10-09

**Authors:** Ramachandran Adhavan, Kuppusamy Selvam, Palanisamy Prakash, Peraman Manimegalai, Dharmalingam Kirubakaran, Muthugounder Subramanian Shivakumar

**Affiliations:** a Department of Botany, Periyar University, Salem, India; b Department of Biotechnology, Periyar University, Salem, India

**Keywords:** *Eranthemum roseum*, Photocatalytic dye degradation, Larvicidal, AO/EtBr staining, Anti-Cancer, Anti-inflammatory, Hypoglycemic, Antioxidant

## Abstract

Synthesis of metal oxide nanoparticles using medicinal plants increasing rapidly due to its eco-friendly to environment. In this study Zinc oxide nanoparticle is synthesized using the leaf extract of plant *E. roseum.* Synthesized NPs was characterized using UV- Vis Spectroscopy analysis where the peak observed at 374 nm with band gap of 2.5 eV, FTIR and XRD analysis validate pure hexagonal structure, Spherical shape of NPs was confirmed by SEM with EDX analysis and main compounds are zinc 75 % and oxygen 22 %. Transmission Electron Microscopy Analysis confirms the oval shaped ZnO NPs Biological activity of *E. roseum* ZnO NPs such as antioxidant assay DPPH, ABTS, hydroxyl radical activity shows good inhibition against free radicals. The In-vitro Hypoglycemic effects has maximum inhibition of 96 % on α- amylase activity and 87 % on α- Glycosidase activity. Anti-inflammatory activity recorded 93 % maximum inhibition at Albumin denaturation assay and 75 % at Membrane stabilization assay. *E. roseum* ZnO NPs shows 67.79 % on HepG2 Anti-proliferative cells line. AO/EtBr staining assays confirms the apoptosis effect. Larvicidal activity shows highest mortality of 98.44 % on species *C. quinquefasciatus.* Photocatalytic dyedegradation of Methylene blue dye shows 65 % of dye degradation.

## Highlights

• This study confirmed the green synthesis of ZnONPs derived from *Eranthemum roseum.*
• The synthesized ZnONPs were characterized through UV, FTIR, XRD, SEM with EDAX and TEM analysis.
• The green-synthesized ZnONPs exhibit remarkable antibacterial, antioxidant, anti-inflammatory and antidiabetic properties.
• The anticancer potential of synthesized ZnONPs was assesed on HepG2 cellS .

## Graphical Abstract

**Figure:**
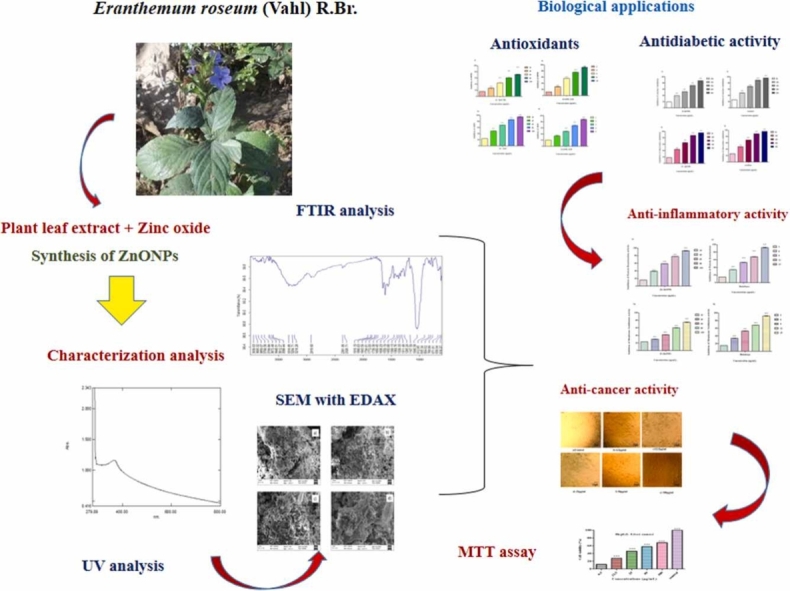

## 1 Introduction

Nanoparticles have proven to be highly valuable due to their unique properties. They can effectively pass through biological barriers and accumulate in diseased tissues, allowing for targeted detection and treatment of single diseased cells. The field of nanotechnology in medicine aims to revolutionize diagnosis and treatment methods [1]. Researchers have been actively investigating low-cost and low-environmental impact approaches for developing well-characterized NPs. These alternative methods serve as substitutes for traditional synthetic processes [2]. One particularly promising technique is the synthesis of NPs using organisms. Different plant parts leaf, root, fruit, seed, bark, flower and whole plant have been utilized for nanoparticle synthesis. NPs synthesized using plant extracts are more stable and have a faster production rate compared to those synthesized using microorganisms [3].

The antioxidant involved from the oxidative stress, caused by free radicals, is implicated in numerous diseases and disorders, including cancer, diabetes, cardiovascular diseases [4]. Free radicals are generated both internally during metabolic activities and externally from various sources such as medications, smoking, and exposure to radiation. Consuming antioxidant-rich foods can help protect the body from the harmful effects of free radicals. Plant extracts and metal oxide NPs are specially focussed and analysed because of their antioxidant potential [5]. Diabetes mellitus is a chronic disease characterized by metabolic disorders in pancreatic β-cells, resulting in hyperglycaemia. There are two main types of diabetes such as type 1 diabetes mellitus, which is caused by a deficiency of insulin production by the pancreas and type 2 diabetes mellitus, which is characterized by insulin resistance despite sufficient insulin production [6]. Recent years, Inflammation is a pathological condition that occurs during injury or infection and aims to restore tissue microenvironment and cellular homeostasis. Nanotechnology has opened up new avenues to detect diseases in primary stage and makes treatment easier and more effective for inflammatory disease [7]. Nanoparticles are loaded by hydrophilic polymers to achieve its absorption and solubility. In case of acute inflammatory this loaded properties of nanoparticles which provides rapid action to antipyretic/ analgesic, anti-inflammatory effect [8]. Liver cancer, specifically hepatocellular carcinoma, is a significant cause of morbidity and mortality worldwide. Zinc oxide nanoparticles are being studied for its potential in curing diabetes mellitus and its associated complications [9]. These nanoparticles have the ability to deliver zinc ions, which play a crucial role in glucose metabolism and homeostasis. Diabetes is a metabolic disorder and a leading cause of death worldwide [10]. In Asia Acanthaceae family is noteworthy due to its distribution throughout the continent. Especially in Indian subcontinent Acanthaceae have distribution of more significant species [11]. *Eranthemum* genus belongs to Acanthaceae family has 104 species which are mostly perennial flowering plants and used as ethno medicine around the world due to its reported gastro protective, antinflammatory, antioxidant and various medicinal properties [12].

According to the available data, there have been no reports of the synthesis of ZnO nanoparticles using *Eranthemum roseum* aqueous leaf extract as a reducing agent in the absence of external reducing and capping agents. We effectively synthesized *Er*- ZnO nanoparticles in this investigation. The antibacterial, antioxidant, anti-inflammatory, antidiabetic, and anticancer properties of these nanoparticles were remarkable. This remarkable discovery suggests that these nanoparticles have the potential to be used in the treatment of cancer and environmental studies, particularly in the context of larvicidal activities. These findings provide avenues for the implementation of *Er*- ZnO nanoparticles in a variety of practical applications.

## 2 Experimental methods

### 2.1 Collection and extraction of Plant materials

*E. roseum* plants are collected in the regions Kalvarayan hills (11°443´32.3´N, 78°18´00.3´E) present in Kallakurichi district, Tamil Nadu, India. During the collection process, we gathered plant samples that included both vegetative and reproductive parts, such as flowers, leaves, and stems, while ensuring that the habitat remained undisturbed. Collected plant samples are undergone authentication at Botanical Survey of India, Coimbatore. Plant material was identified as *Eranthemum roseum* with in BSI, Coimbatore specimen number - BSI/SRC/5/23/2022/Tech/513. The collected leaves were carefully rinsed with distilled water and washed with running tap water in order to remove any impurities. Subsequently, the washed leaves were dried in room temperature. The dried leaves were ground into powdered form using an electric blender. The resulting was stored in an airtight container to maintain its quality. In order to prepare leaf extract, 10 g of powdered *E. roseum* was added to 100 mL distilled water the mixture was heated and boiled at 60°C for 25 min. After boiling, the extract was purified using whatman No. 1 filter paper to remove remaining solid particles. Purified extract was then stored at a temperature of 4°C for future use.

### 2.2 Synthesis of ZnONPs

To prepare 2 M zinc acetate aqueous solution mixture of 50 mL Deionized water and 5 mL plant leaf extract is added. Subsequently pH 13 was adjusted by adding 2 M NaOH. Mixture was kept undisturbed for 2 hrs. Formation of pale white precipitate, confirms synthesized zinc acetate and white precipitate was washed in distilled water in order to remove impurities. Following that, it was further washed with ethanol to ensure the elimination of contamination. Precipitate was centrifuged at 5000 rpm for 15 min to separate solid particles. The resulting precipitate was then dried at a 60°C. The dried particles was subjected to heat in muffle furnace at 350°C for duration of 3 hours. This heating process helped to further stabilize the zinc acetate precipitate. Through these steps, 2 M zinc acetate aqueous solution was prepared, and the synthesis of the white precipitate was successfully achieved [13].

### 2.3 ZnONPs characterization

#### 2.3.1 UV-Vis Spec analysis

Shimadzu UV- Spectrophotometer (Model UV- 1800) used to conform formation ZnONPs through measuring the UV-Visible spectra within the range of 200–600 nm

#### 2.3.2 FTIR analysis

Functional group of synthesized ZnONPs is analysed using FTIR - Perkin Elmer system 200 USA. ZnONPs were transformed into pellets KBr, and the spectra were recorded within wavelength range of 400–4000 cm^−1^.

#### 2.3.3 X-Ray diffraction analysis

Average structure of synthesized *Er*-ZnONPs was analysed using XRD - XPERT PROPAN, PHILIPS, USA. High intensity peak is calculated using Debye- Scherrer equation D=Kλ/β (Cosθ).

#### 2.3.4 SEM with EDAX analysis

ZEISS SEM is used to find shape and size of ZnONPs. Particle size are calculated and measured by IMAGE J Software. EDAX – Quorum technologies is employed in mapping elemental elements dispersion X-ray identification.

#### 2.3.5 TEM analysis

TEM model JEOL – JEM – 2100 is employed in analysing the size and structure ZnONPs.

### 2.4 *In-vitro* antioxidant activity

#### 2.4.1 DPPH assay

The DPPH activity was assessed by [14]. ZnONPs is added to test tubes on various concentrations 25–125 µg/ mL. 0.2 M DPPH 1 mL was added in test tubes and incubated for 30 min. Yellow colour change from purple is noted and under UV –Vis spec sample absorbance was measured under 517 nm. Vitamin C is compared as standard.

$$\mathrm{Inhbition}\;\%\;\mathrm{of}\;\mathrm{DPPH}=\mathrm{Absorption}\left[\frac{\mathrm{Control}-\mathrm{Sample}}{\mathrm{Control}}\right]\times 100$$

#### 2.4.2 ABTS assay

ABTS activity was employed according to [14]. Various concentrations of ZnONPs extract (25–125 µg/mL) is poured in test tubes. 1 mL 7mM ABTS solution is poured in each test tubes with 2.45 mM potassium persulfate and mixed and incubated for ten minutes. Absorbance of mixture is absorbed under 734 nm in UV- Vis spec. Vitamin C is compared as standard.

$$\mathrm{Inhibition}\;\%\;\mathrm{of}\;\mathrm{ABTS}=\mathrm{Absorption}\left[\frac{\mathrm{Control}-\mathrm{Sample}}{\mathrm{Control}}\right]\times 100$$

#### 2.4.3 Hydroxyl scavenging assay

Hydroxyl scavenging assay is conducted by methods [15]. In this test FeSO_4_ 1 mL 1.5 nm was mixed with 0.7 mL 6 mM of H_2_O_2_ followed by 0.3 mL 20 mM sodium salicylate 0.3 mL of a 20 mM C_7_H_5_NaO_3_ solution was added to this mixture. The 1 mg/mL ZnONPs was diluted with different concentrations of 25, 50, 75, 100 and 125 µg/mL deionized water. The resulting mixture was left undisturbed in 37ºC for period of 30 min. Vitamin C is used as control absorbance is measured at 562 nm

$$\mathrm{Inhibition}\;\%\;\mathrm{of}\;\mathrm{Hydroxyl}\;\mathrm{scavenging}=\mathrm{Absorption}\left[\frac{\mathrm{Control}-\mathrm{Sample}}{\mathrm{Control}}\right]\times 100$$

### 2.5 Hypoglycemic effects

#### 2.5.1 α- Amylase inhibition

***α*** – amylase assay is conducted by following [16]. Different concentration ZnO NPs solution is added with 1 mL ***α*** – amylase and 2 mMphosphate buffer 500 μl. The mixture is incubated for 20 min soluble starch 500 μl is added with the resulting mixture incubated for 5 min and 1 mL DNSA is added to test tubes with mixture and kept in water bath for 5 min. The absorbance of mixture is measured under 540 nm in UV- Vis spectrophotometer.

$$\mathrm{Inhibition}\;\%\;\mathrm{of}\;\alpha -\mathrm{Amylase}=\mathrm{Absorption}\left[\frac{\mathrm{Control}-\mathrm{Sample}}{\mathrm{Control}}\right]\times 100$$

#### 2.5.2 α-Glucosidase inhibition

This assay is performed by 20 µL According to the procedure outlined by [16] 20 µL α- glucosidase is taken in test tubes and 0.1 M phosphate buffer 5 mg/mL is added while the 6.9 pH is maintained. Resulting mixture is incubated for 10 min in 25ºC. ZnO NPs was made in different concentration then added to mixture and incubated for 5 min under 25ºC. Resulting mixture absorbance is measured at 405 nm in UV – Visible spectrophotometer. Inhibition percentage of α- glucosidase is calculated by following formulae.

$$\mathrm{Inhibition}\;\%\;\alpha -\mathrm{glycosidase}=\mathrm{Absorption}\left[\frac{\mathrm{Control}-\mathrm{Sample}}{\mathrm{Control}}\right]\times 100$$

### 2.6 *In-vitro* anti -inflammatory analysis

#### 2.6.1 Albumin denaturation assay

This test performed as previously described by [17]. At different concentrations (50–250 µg/mL) of *Er*-ZnONPs added along 0.45 mL of 15 % egg albumin serum 0.5 mL dis H_2_O and mixed well. pH maintained 6.3 by adding 1 N HCL and kept undisturbed for 5 min. After 20 min incubation at 37ºC mixture was heated for 5 min at 57ºC rapidly cooled for 5 min. Finally 2.5 mL PBS was added. Then absorbance was recorded under UV-Vis spectrophotometry and aspirin as standard.

$$\mathrm{Inhibition}\;\%\;\mathrm{of}\;\mathrm{Albumin}\;\mathrm{denaturation}=\mathrm{Absorption}\left[\frac{\mathrm{Control}-\mathrm{Sample}}{\mathrm{Control}}\right]\times 100$$

#### 2.6.2 Membrane stabilization assay

The experiments were conducted according to [17] protocol. Collecting blood cells and placing them in 2 mL containers. After 5 min, an equal volume of Alsever's solution containing 2 % dextrose, 0.8 % sodium nitrate, 0.5 % citric acid, and 0.42 % NaCl was added along with 2 mL of distilled water and the mixture was centrifuged at 3000 rpm. With isosaline solution the cells were packed and 10 % (v/v) suspension at 4ºC. This cell suspension was then added to test containers containing different concentration ZnONPs 50, 100, 150, 200 and 250 µg/mL. By adding 0.5 mL 10 % human red blood cell membrane stabilization suspension, 2 mL hyper saline, 1 mL Phosphate-buffered solution in each test tube. After incubating the mixtures at 37°C for 30 minutes, they were centrifuged at 3000 rpm for 20 min. The supernatant solution was collected, and the haemoglobin concentration determined by spectrophotometry at 560 nm. Aspirin was used as a comparison standard. In this experiment, the ability of ZnONPs extracts to stabilize cell membranes was assessed. The test results provided insight into the extract's ability to prevent membrane injury to red blood cells, with aspirin serving as a comparison compound.

$$\mathrm{Inhibition}\;\%\;\mathrm{of}\;\mathrm{Membrane}\;\mathrm{stabilization}=\mathrm{Absorption}\left[\frac{\mathrm{Control}-\mathrm{Sample}}{\mathrm{Control}}\right]\times 100$$

#### 2.6.3 Culturing HepG2 cell and MTT assay

HepG2 liver cancer cells line is cultured in plates with 96 well and in conical flask hank salts 10 % fetal bovine serum in DMEM is added. Then 2 mM/ L glutamine and aminoacids added and at 37°C incubated for 24hrs. Different concentration of *Er* - ZnO NPs is added resulting mixture is absorbed in 570 nm in microplate reader [18].

$$\mathrm{Cell viability}\;\%=\left[\frac{\mathrm{Optical}\;\mathrm{density}\;\mathrm{of}\;\mathrm{sample}}{\mathrm{Optical}\;\mathrm{density}\;\mathrm{of}\;\mathrm{control}}\right]\times 100$$

### 2.7 EtBr /AO staining

In Dulbecco’s Eagle Medium HepG2 cells was seeded in 96 well plates followed by one night full of incubation. By using Phosphate Buffered Saline the cells were washed and treated with sample seeded in the medium and incubated for 37°C in 5 % CO_2_ incubator for 24 h. While the incubation is over the medium is treated with ethidium bromide, acridine orange 10 µL of 1 mg/mL. At last the cells have examined under fluorescent filter in Fluorescence microscope [19].

### 2.8 Larvicidal bioassay

The Larvae was collected from the nearby paddy field and water stagnant area. The collected larvae was observed under electronic microscope in order to observe the morphological characters to identify the species of larvae. Toxicity of ZnO NPs was carried out in 4^th^ instar larva in *Aedes aegypti, Anopheles stephensi* and *Culex quinquefasciatus*. 25 larva of each species was added in glass containing 200 mL dechlorinated water. The synthesised nanoparticles in different concentrations at (5–25 mg/L) was added in all cups while double distilled water is serves as control. Death of larva was observed after 24 hrs [20]. The mortality percentage of all lethal concentration at LC_50_ and LC_90_ was calculated using software SPSS Version 20. 0.

### 2.9 Photocatalytic dye degradation

The methylene blue dye was photocatalytically degraded using ZnONPs in the presence of sunlight. The experiment was carried out in a glass beaker with 100 mL of 20 ppm methylene blue dye and 50 mg of ZnONPs, mixed in the presence of sunlight on a sunny day. Samples were gathered during the procedure, and the UV-Vis spectrophotometry was used to measure the rate of deterioration at the absorbance peak, which is between 300 and 800 nm, followed by [21] with minor modifications.

$$\mathrm{Degradation}\;\%=\left[\frac{\mathrm{Ci}-\mathrm{Cf}}{\mathrm{Ci}}\right]\times 100$$

Where Ci is the methylene blue dyes beginning concentration. The methylene blue dyes final concentration is known as Cf.

### 2.10 Statistical data analysis

All experiments are done in triplicate n = 3. Orign 8 version 8.0725 is used in interpreting the research data’s in graphs. Prism software version 5.2 used for analysis of Mean ± Standard deviation and asterisk (***) indicates significance differences of sample and control in one - way ANOVA (P < 0.001).

## 3 Results and discussion

### 3.1 UV spectroscopy analysis

**Fig. 1:**
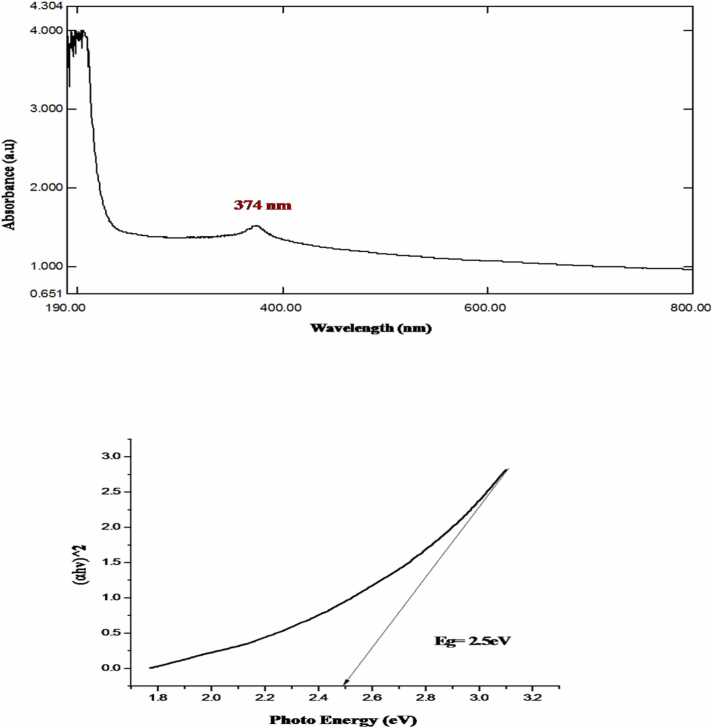
a) Uv –Vis spectrum of biosynthesized *E. roseum* ZnONPs b) Plot of (αhv)^2^ against to calculate band gap of *E. roseum* ZnONPs.

Biosynthesis of zinc oxide nanoparticles wavelength were assessed by UV–visible spectrophotometry. UV visible spectrum wavelength was set to 200–800 nm, with a peak observed at 374 nm illustrated in Fig. 1. The similar results from the ZnONPs synthesis using extracts from *Cyathocline purpurea* leaf extract leaves were reported by [22] with the UV spectrum length being detected from 371 nm. Similar results were obtained by [23] using *Trigonella foenum- graecum* leaf extract in ZnO nanoparticle synthesis at 371 nm. According to [24] more results from the synthesis of ZnONPs using extracts from *Lablab purpureus* leaves showed that the UV spectrum length at 371 nm. It was further shown that zinc oxide (ZnO) plays a major part in photocatalysis because of its high light-utilization coefficient. Availability of oxygen vacancies, which may facilitate the transfer of electrons from the Valence Band (VB) to the Conduction Band (CB), may be the cause of the bandgap of 2.5 eV (Fig. 1).

### 3.2 FTIR analysis

**Fig. 2:**
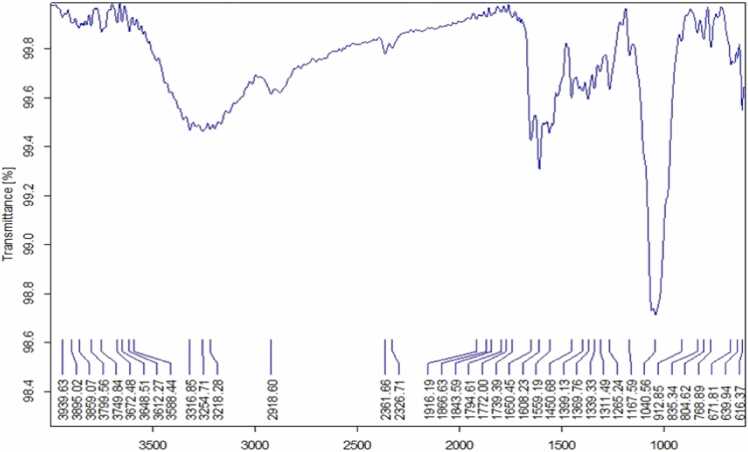
FTIR analysis of biosynthesized *E. roseum* ZnONPs.

The retention range of the synthesized ZnONPs of *E. roseum* shows the presence of various functional groups 648.93 cm^−1^ (Strong C-Br Stretching) denotes Halo compounds, 839.11 (Medium C=C Bending) denotes the presence Alkenes, 1049.87 (Strong CO–O–CO stretching) denotes Anhydride, 1345.73 (Medium O-H bending) denotes Phenol, 1405.54 (Strong O-H bending) denotes Alcohol, 2163.02 (Strong S-CΞN stretching) denotes Thiocynate (Medium N–H stretching) denotes Aliphatic primary amine presented in Fig. 2 and Table 1. The presence of O-H stretching conforms the ZnONPs and similar results in FT-IR studies of *Laurus nobilis* synthesized ZnONPs phytochemicals like alcohols, phenols, amines interact to zinc and responsible for ZnONPs synthesis [25].

**Table 1:** FTIR peak values and functional groups *E. roseum* ZnONPs.

**S. No**	**Wave number (Cm**^**−1**^**)**	**Intensity**	**Bond responsible**	**Functional** **Groups**
1	648.93	Strong	C-Br Stretching	Halo compounds
2	839.11	Medium	C=C Bending	Trisubstituted
3	929.65	Strong	S=O	Sulfoxide
4	1020.95	Medium	C-N stretching	Amine
5	1049.87	Strong	CO–O–CO stretching	Anhydride
6	1345.73	Medium	O-H bending	Phenol
7	1405.54	Strong	O-H bending	Alcohol
8	1554.04	Medium	C–H	Alkanes
9	2163.02	Strong	S-CΞN stretching	Thiocyanate
10	3357.55	Medium	N–H stretching	Aliphatic primary amine

### 3.3 XRD analysis

**Fig. 3:**
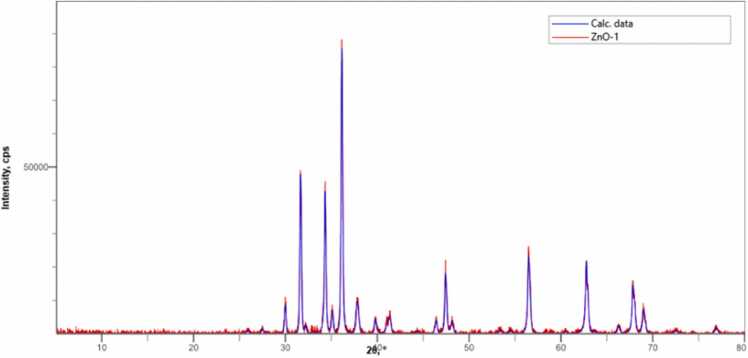
The XRD pattern analysis of biosynthesized *E. roseum* ZnONPs*.*

XRD of *E. roseum* of ZnONPs observed values such as 31.66, 34.38, 36.13, 47.55, 56.48, 62.76, 67.66 and 69.15 observed presented in Fig. 3 value responsible for planes 100, 002, 101, 102, 103, 200, 112 resulting the pure hexagonal structure of ZnO NPs (JCPDS No. 00–036–1451) [26]. Obtained similar results the planes (100), (002), (101), (102), (110), (103), (200), (112) and (201) responsible for the structure hexagonal on the XRD analysis of *Hyphaene thebaica* and in *Papaver somniferum* ZnONPs the XRD analysis shows (100), (002), (101), (102), (110), (103), (200), (112), (201) crystal planes which conforms the Hexagonal shape of ZnONPs [27].

### 3.4 SEM EDAX analysis

**Fig. 4:**
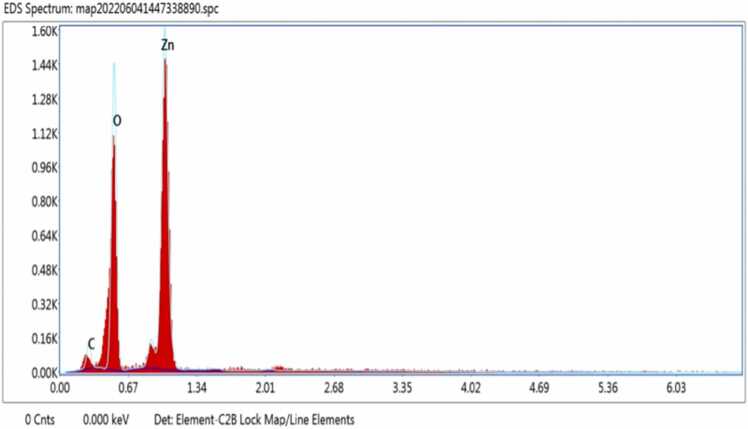
EDX analysis of *E. roseum* ZnONPs.

**Fig. 5:**
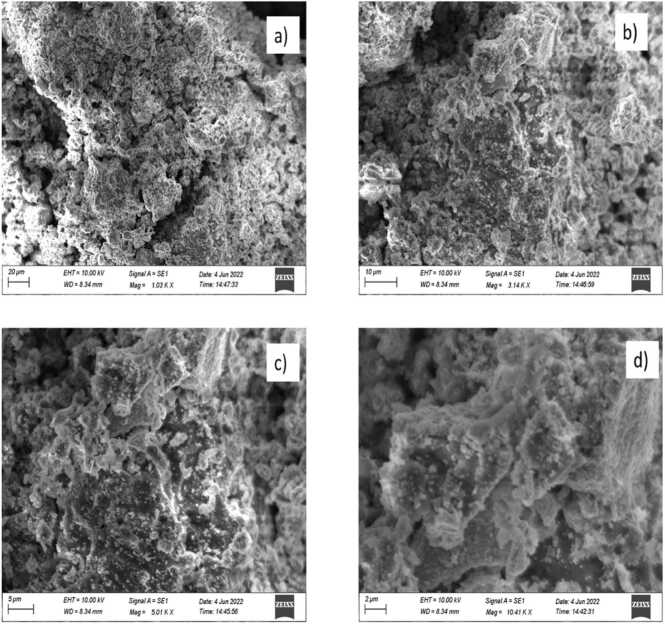
SEM analysis of *E. roseum* ZnONPs.

*E. roseum* ZnONPs, SEM image showed a spherical shape in Fig. 4, Fig. 5. SEM element analysis of synthesized ZnONPs was spherical morphology form *Nephrolepis exaltata* extract [28]. While comparing with ZnONPs synthesised using *Hardwickia binata* leaf extract also obtained similar Spherical shape [29]. The element analysis of synthesized ZnONPs by EDAX were also done *E. roseum* an leaf extract assisted ZnONPs, the main compounds are zinc 75 % and oxygen are 22 %.

### 3.5 TEM analysis

**Fig. 6:**
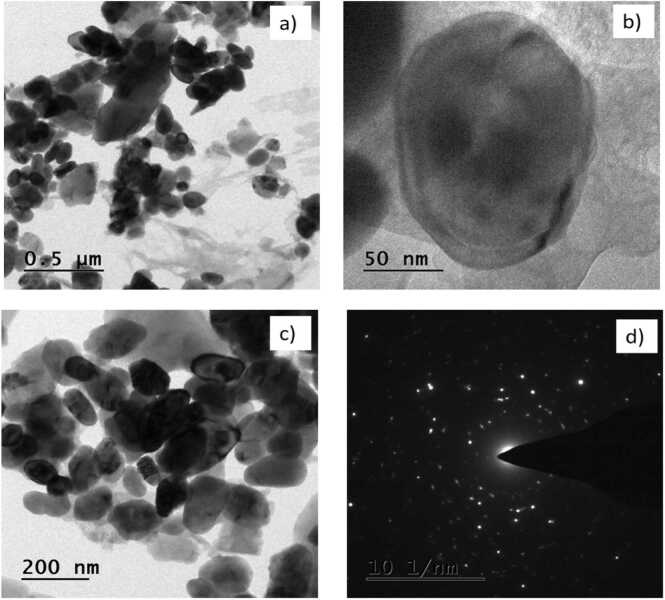
TEM analysis of synthesized *E. roseum* ZnONPs at different magnifications Selected Area Electron Diffraction (SAED) pattern.

ZnO compounds are indicated by the TEM and SAED pattern. Following TEM investigation, the size of the nanoparticles was examined (Fig. 6). TEM images of *Coleus forskohlii* ZnO nanoparticle conforms the oval shape [30]. [31] Were reported the TEM investigation from leaf extract of *Juglans regia* ZnO NPs conforms oval shape of particles.

### 3.6 Antioxidant analysis

#### 3.6.1 DPPH assay

**Fig. 7:**
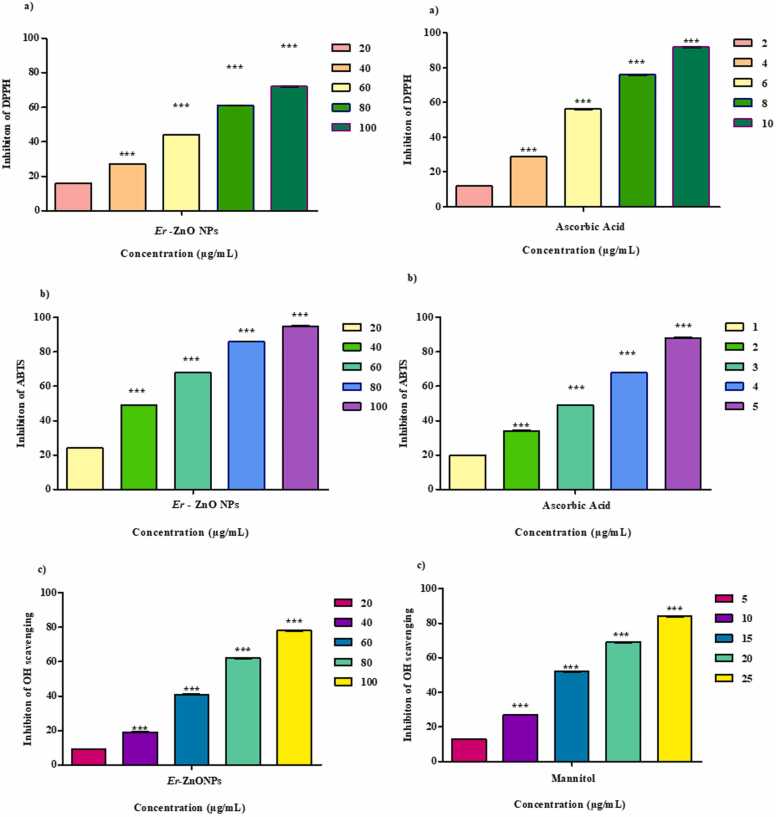
Antioxidant potential of *E. roseum* ZnONPs acid a) DPPH radical scavenging activity b) ABTS^+^ radical scavenging activity, Standarad Ascorbic acid and c) Hydroxyl scavenging activity, Standard Mannitol.

Synthesized ZnONPs DPPH analysis activity resulting the maximum level of inhibition at concentration 100 µg/mL^−1^ (92 %) while minimum level of inhibition at 20 µg/mL (12 %) while IC_50_ is 67 µg/mL illustrated in Fig. 7. Similar report (78.52 %) inhibition was showed in DPPH analysis of ZnO nanoparticle in *Drynaria quercifolia* [32]. Similar report (75 %) inhibition was reported in DPPH activity of ZnO nanoparticle in *Solanum lycopersicum* fruit extract [33].

#### 3.6.2 ABTS assay

The ABTS analysis on ZnONPs obtained maximum level of inhibition on 100 µg/mL (95 %) followed by the minimum inhibition recorded in the 20 µg/mL (24 %) while IC_50_ recorded as 43.9 µg/mL showed in Fig. 7. Same level of inhibition of (95 %) was recorded in the ABTS analysis on ZnONPs synthesized in *Punica granatum* [34]. Similarly, significant (91.91 %) ABTS analysis on ZnO NPs using *Saponaria officinalis* [35].

#### 3.6.3 OH scavenging assay

OH scavenging of synthesized nanoparticles shows 78 % inhibition at (100 µg/mL) and minimum inhibition 9 % (20 µg/mL) have IC_50_ 69.06 µg/mL represented in Fig. 7. Hydroxyl radical scavenging activity of Zinc NPs conducted [36] shows similar results of 50.9 %, 48.73 % of inhibition. OH radicals affects all the biological molecules which are formed in hydrogen peroxide known as Fenton reaction [37].

### 3.7 In - vitro hypoglycemic activity

#### 3.7.1 α - amylase activity

**Fig. 8:**
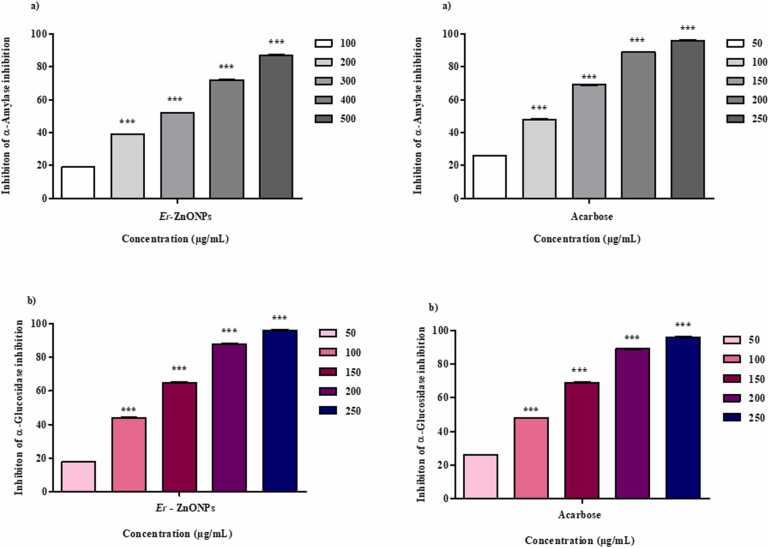
α - amylase and α –glycosidase activity of *E. roseum* ZnONPs and standard Acarbose.

The α - amylase activity has least inhibition recorded in 50 µg/mL (26 %) while highest inhibition recorded in 250 µg/mL (96 %) and IC_50_ value 106.9 % µg/mL (Fig. 8). Similarly [38] reported that the 51 % of inhibition of synthesized zinc nanoparticles from immobilization of α-amylase. Another report were found from zinc oxide from *Myristica fragrans* fruit extract [39].

#### 3.7.2 α - Glucosidase activity

α-Glucosidase activity of of ZnONPs has lowest inhibition 100 µg/mL (19 %) the highest inhibition as 87 % at 500 µg/ mL which has IC_50_ of 386.27 µg/mL (Fig. 8). [16] Found the 16.3 µg/mL α-glucosidase inhibition by ZnONPs synthesized using a *Tabemaemontana heyneana*. Another report were confirmed from ZnONP leaf extract of *Aquilegia pubiflora* shows 22.69 % [40]. Similar results were found from *Myristica fragrans* using zinc nanoparticles had 65.21 µg/mL.

### 3.8 In- vitro Anti- inflammatory activity

#### 3.8.1 Albumin denaturation assay

**Fig. 9:**
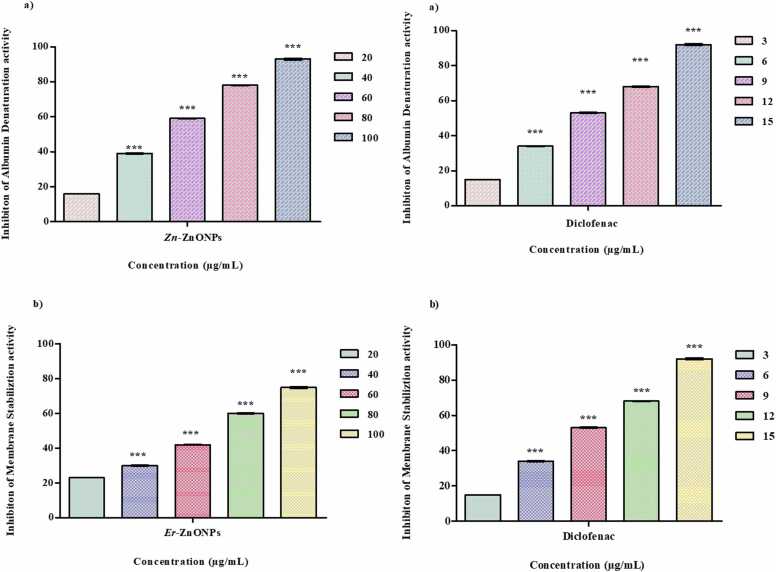
Anti-inflammatory activity of *E. roseum* ZnONPs and standard Diclofenac a) Albumin denaturation assay b) HRBC membrane stabilization assay.

Albumin denaturation assay of synthesized ZnONPs to identify the inhibition level of inflammatory at Maximum inhibition 93 % at 100 µg/ mL minimum level of inhibition 16 % at 20 µg/mL with IC_50_ 53.02 µg/mL^−1^ showed in (Fig. 9). Similar results were reported by [41]. Another report [18] were analysed from spinach mediated zinc nanoparticles synthesis had 39.1 µg/mL [42]. Recent report were analysed from green tea using ZnONPs synthesis had 87 % of inhibition [43].

#### 3.8.2 Membrane Stabilization assay

Albumin denaturation assay of synthesized ZnONPs to identify the inhibition level of inflammatory at Maximum inhibition 75 % at 100 µg/ mL, minimum inhibition 23 % at 20 µg/mL with IC_50_ value 83.3141 µg/mL (Fig. 9). [44] Stated similar result on African basil and black tulsi ZnONPs with 66 % and 72 % of inhibition level at membrane stabilization assay.

### 3.9 Anti-proliferative effect of HepG2 Cells

**Fig. 10:**
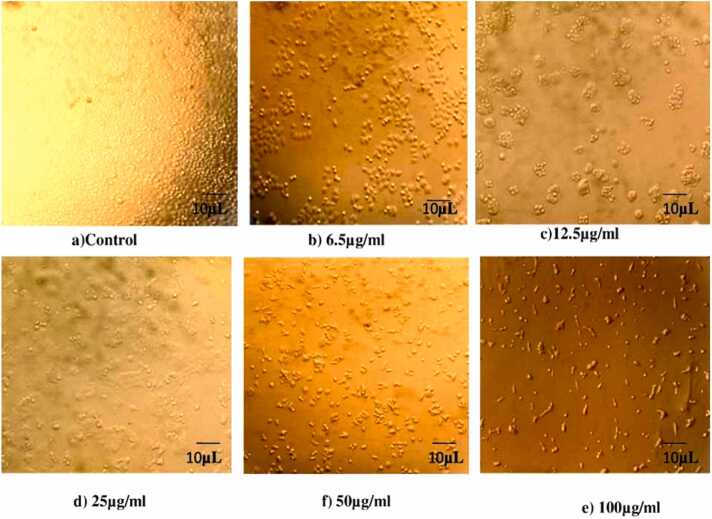
Treated and untreated cells HepG2 cells against of *E. roseum* ZnONPs.

**Fig. 11:**
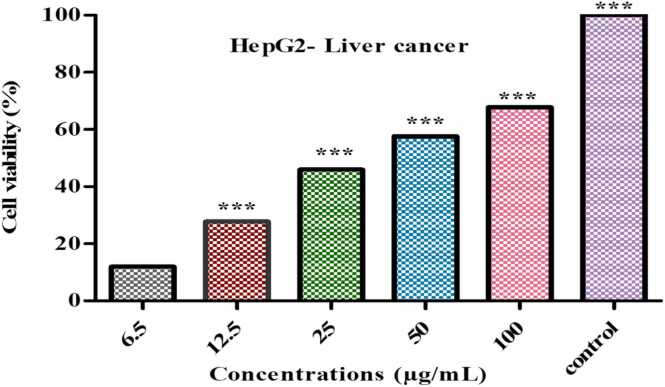
Anti-proliferative activity of *E. roseum* against HepG2 liver cancer cell line.

Anti-proliferative effect of ZnONPs has showed the IC_50_ value at 30.39 µg/mL and 100 µg/mL high inhibition of (67.79 %) least inhibition 6.5 µg/mL (11.92 %) illustrated in Fig. 10, Fig. 11. Similar report were analysed from zinc oxide nanoparticles using *Lepidium sativum* has 30 % of inhibition at the concentration 200 µg/mL [45]. Other reports were confirmed from ZnO nanoparticles using *Pandanus odorifer* has 62 % of inhibition [46].

### 3.10 Apoptotic effects exploration by AO / EtBr staining

**Fig. 12:**
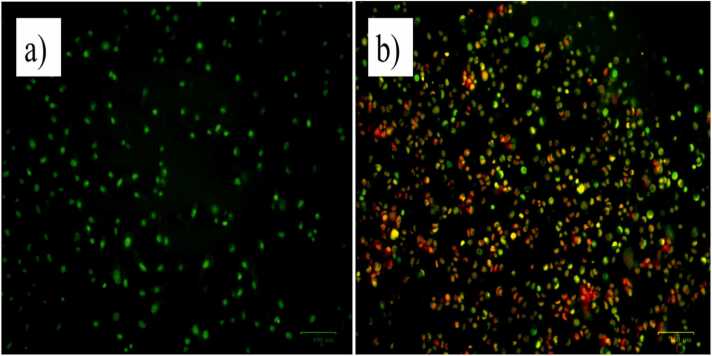
Fluorescence microscopic analysis of AO/EtBr assay a). Untreated cell b) Treated with 70 μg/mL of *E. roseum* ZnONPs of HepG2 Cell line.

HepG2 cells exposed to ZnONPs showed alteration in cell morphology while untreated control cells showed no morphological changes. Microscopic observation suggests that, diffusion of acridine orange (AO) into the cell membrane, the stable and viable cells appeared in the orange colour due to a nuclear shrinkage and blurring. Apoptotic cells seen orange colour cells. However, to loss of cell membrane caused by an integrity of ZnONPs which causes cytotoxicity, the necrotic cells were transformed into red colour represented in Fig. 12. Interestingly, in cells treated with exact aqueous leaf ZnONPs further apoptosis was observed [47].

**Fig. 13:**
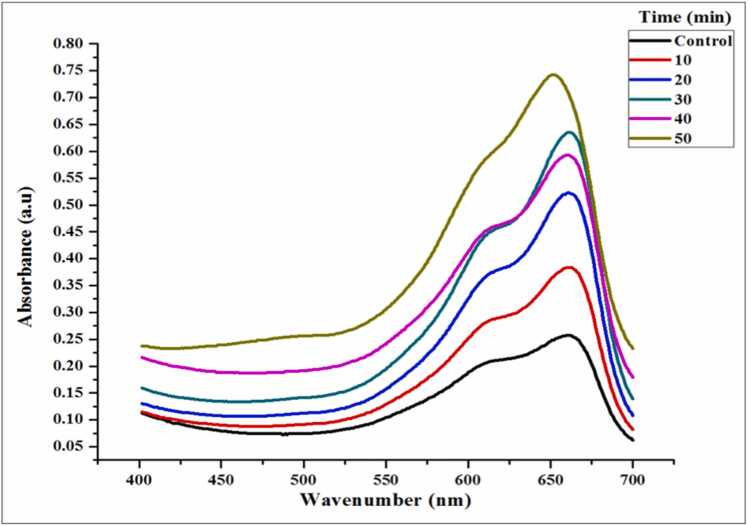
Photocatalytic dye degradation of Methylene blue dye of *E. roseum* ZnONPs.

### 3.11 ZnO NPs Larvicidal activity

*Er*- ZnONPs was make in several concentration (5, 10, 20, 25 µg/mL) in order to explore the larvicidal activity against the selected represented in Table 2. The ZnONPs revealed the strong larval mortality in *A*. *aegypti* with highest LC_50_ 5.80 and LC_90_ 24.08) *A. stephensi* (LC_50_ 3.35 with LC_90_ 23.06) and *C. quinquefaciatus* (LC_50_ value as 02.87 LC_90_ value as 18.15). The ZnONPs synthesized from *E. roseum* has very good effect in controlling the mosquito larvae and helps in preventing fatal disease in humans [48].

**Table 2:** Larvicidal activity of ZnONPs of *E. roseum* against *Aedes aegypti, Anopheles stephensi* and *Culex quinquefasciatus.*

**Time Exposure** **Hours**	**Mosquito** **Species**	**Concentration (mg/l)**	**Mortality%**	**LC**_**50**_**(mg/l)** **(LCL-UCL)**	**LC**_**90**_**(mg/l)** **(LCL-UCL)**	**χ**^**2**^
24	*A. aegypti*	25	93.33±2.87	5.80 (1.07–08.58)	24.08 (20.73–30.18)	2.39
		20	81.66±1.67			
		15	71.66±2.87			
		10	63.33±0.50			
		5	48.66±2.87			
		0	00.00±0.00			
24	*A.* *stephensi*	25	91.66±2.87	3.35 (3.22–06.79)	23.06 (19.64–29.56)	1.76
		20	86.66±1.66			
		15	76.66±1.66			
		10	68.33±2.87			
		5	53.33±3.33			
		0	00.00±0.00			
24	*C. quinquefasciatus*	25	98.66±2.87	02.87 (02.39–05.82)	18.15 (15.73–22.08)	3.46
		20	91.66±1.66			
		15	81.66±0.50			
		10	73.33±1.66			
		5	58.33±1.66			
		0	00.00±0.00			

### 3.12 Photocatalytic dye degradation of methylene blue dye

ZnONPs photocatalytic ability was examined using aqueous solutions and methylene blue dye under natural sunlight. The varying absorption spectra associated with the methylene blue dye degradation were observed at different time intervals (0–50 min). After 50 min of exposure of mixture with *E. roseum* ZnONPs and methylene blue dye the decolourization occurs which shows 65 % of dye degradation. Similarly, synthsized ZnO using *Sambucus ebulus* extract of showed 80 % of degradation in 200 min [49]. In another report showed 94 % of degradation using *Solanum trilobatum* leaf exract [50].

## 4 Conclusion

Zinc oxide nanoparticles are synthesized from *Eranthemum roseum* leaf extract which is eco-friendly, simple and non-toxic. The synthesised ZnONPs under gone characterization in which the UV spectroscopy conforms the synthesized zinc at 371 nm. The planes represented in XRD analysis conforms the Hexagonal structure NPs. The SEM analysis shows spherical shape and EDAX indicates 75 % of zinc in *Er*-ZnONPs. TEM analysis shows the oval structure of synthesized *Er*-ZnONPs. Biomedical applications such as AO/EtBr staining, Anti-proliferative, Anti-inflammatory, Antioxidant, Hypoglycemic shows good inhibition level against the disease causing agents due to the combination of zinc oxide and *Eranthemum roseum*. Larvicidal effects in the *Er*-ZnONPs has good inhibition in three different species of mosquito larva. Thus the *Er*-ZnONPs has potential and has ability to play role in biomedical, pharmaceutical and pesticide industries.

## Author Statement

Author Agreement Statement we the undersigned declare that this manuscript is original, has not been published before and is not currently being considered for publication elsewhere. We confirm that the manuscript has been read and approved by all named authors and that there are no other persons who satisfied the criteria for authorship are not listed. We further confirm that the order of authors listed in the manuscript has been approved by all of us. We understand that the Corresponding Author is the sole contact for the Editorial process. He is responsible for communicating with the other authors about progress, submissions of revisions and final approval of proofs.

## CRediT authorship contribution statement

**Palanisamy Prakash:** Writing – review & editing, Software, Data curation. **Kuppusamy Selvam:** Writing – review & editing, Supervision, Methodology, Formal analysis. **Ramachandran Adhavan:** Writing – review & editing, Writing – original draft, Visualization, Validation, Software, Resources, Methodology, Formal analysis, Data curation, Conceptualization. **Muthugounder Subaramanian Shivakumar:** Writing – review & editing, Visualization, Data curation. **Dharmalingam Kirubakaran:** Writing – review & editing, Visualization. **Peraman Manimegalai:** Writing – review & editing, Software.

## Declaration of Competing Interest

The authors declare that they have no known competing financial interests or personal relationships that could have appeared to influence the work reported in this paper.

## Data Availability

The data that has been used is confidential.
